# Desmodium mottle virus, the first legumovirus (genus *Begomovirus*) from East Africa

**DOI:** 10.1007/s00705-017-3289-1

**Published:** 2017-02-27

**Authors:** Happyness G. Mollel, Peter Sseruwagi, Joseph Ndunguru, Titus Alicai, John Colvin, Jesús Navas-Castillo, Elvira Fiallo-Olivé

**Affiliations:** 1 Instituto de Hortofruticultura Subtropical y Mediterránea “La Mayora”, Universidad de Málaga - Consejo Superior de Investigaciones Científicas (IHSM-UMA-CSIC), Estación Experimental “La Mayora”, 29750 Algarrobo-Costa, Málaga Spain; 2 Mikocheni Agricultural Research Institute, P.O. Box 6226, Dar es Salaam, Tanzania; 3 National Crops Resources Research Institute, Namulonge, P.O. Box 7084, Kampala, Uganda; 4 Natural Resources Institute, University of Greenwich, Kent ME4 4TB, UK

## Abstract

A novel bipartite legumovirus (genus *Begomovirus*, family *Geminiviridae*), that naturally infects the wild leguminous plant *Desmodium* sp. in Uganda, was molecularly characterized and named Desmodium mottle virus. The highest nucleotide identities for DNA-A, obtained from two field-collected samples, were 79.9% and 80.1% with the legumovirus, soybean mild mottle virus. DNA-B had the highest nucleotide identities (65.4% and 66.4%) with a typical non-legumovirus Old World begomovirus, African cassava mosaic virus. This is the first report of a legumovirus in East Africa and extends the known diversity of begomoviruses found infecting wild plants in this continent.

The family *Geminiviridae* comprises seven genera, differentiated based on genome organization, nucleotide sequence identity and biological properties: *Begomovirus*, *Mastrevirus*, *Eragrovirus*, *Curtovirus*, *Turncurtovirus*, *Topocuvirus* and *Becurtovirus* [4, 27]. The genus *Begomovirus* is the largest in the family, with 322 accepted species [5, https://talk.ictvonline.org/ictv_wikis/geminiviridae/m/files_gemini/5120]. Begomoviruses are transmitted by the whitefly *Bemisia tabaci* (Hemiptera: Aleyrodidae) and frequently cause important plant-diseases around the world [21]. Bipartite begomoviruses possess two genome components (DNA-A and DNA-B), which are essential for virus infectivity and the size of each component ranges between 2.5 and 2.8 kb. DNA-A and DNA-B share ~200 nt in the common region (CR), located within the intergenic region, that contains cis elements for replication and control of gene expression. The CR exhibits a high degree of sequence identity between both genome components of bipartite begomoviruses [4]. Based on the phylogenetic analysis of complete nucleotide sequences of DNA-A, begomoviruses can be classified into four lineages, Old World (OW), New World (NW), sweepoviruses and legumoviruses [3].

Legumoviruses, or legume-infecting begomoviruses from the OW, are amongst the most atypical begomoviruses [16]. They are distinct from the numerous legume-infecting begomoviruses that occur in the Americas and in phylogenetic analyses they group in a cluster different from those of OW and NW begomoviruses [3, 10]. The difference between legumoviruses and typical OW begomoviruses could have arisen due to genetic isolation involving either a host-range barrier or lack of movement of whitefly vectors between legumes and non-leguminous plants, thereby preventing genetic exchange between both groups of viruses [24]. The genomes of most legumoviruses are bipartite, although a DNA-B component has not been identified for cowpea golden mosaic virus (CPGMV), Dolichos yellow mosaic virus (DoYMV) and soybean mild mottle virus (SbMMoV) [1, 19]. Little attention has been paid to legumoviruses infecting wild plants. Scarce examples include DoYMV infecting *Lablab purpureus* (sin. *Dolichos lablab*) [19] and horsegram yellow mosaic virus (HgYMV) infecting *Macrotyloma uniflorum* [2] in India, kudzu mosaic virus (KuMV) infecting *Pueraria montana* in Vietnam [13], soybean chlorotic blotch virus (SbCBV) infecting *Centrosema pubescens* in Nigeria [1] and Rhynchosia yellow mosaic virus (RhYMV) infecting *Rhynchosia minima* in Pakistan [15].

In this study, leaf samples of two *Desmodium* sp. (family Leguminosae) plants showing mottle symptoms (Fig. 1) were collected in Kikonge, southwestern Uganda, in March 2015 (00°22.641’ N; 32°11.252’ E [sample UG4], 00°22.640’ N; 32°11.252’ E [sample UG5]). Morphological identification of the plant samples at the genus level was confirmed molecularly by DNA barcoding using chloroplast *rbcL* and *matK* genes [14]. The genus *Desmodium* is composed of about 370 accepted species native to tropical East Asia, Africa and America. Some *Desmodium* species are considered as weeds, although others containing potent secondary metabolites are used in agriculture in push-pull technology [7].

**Fig. 1:**
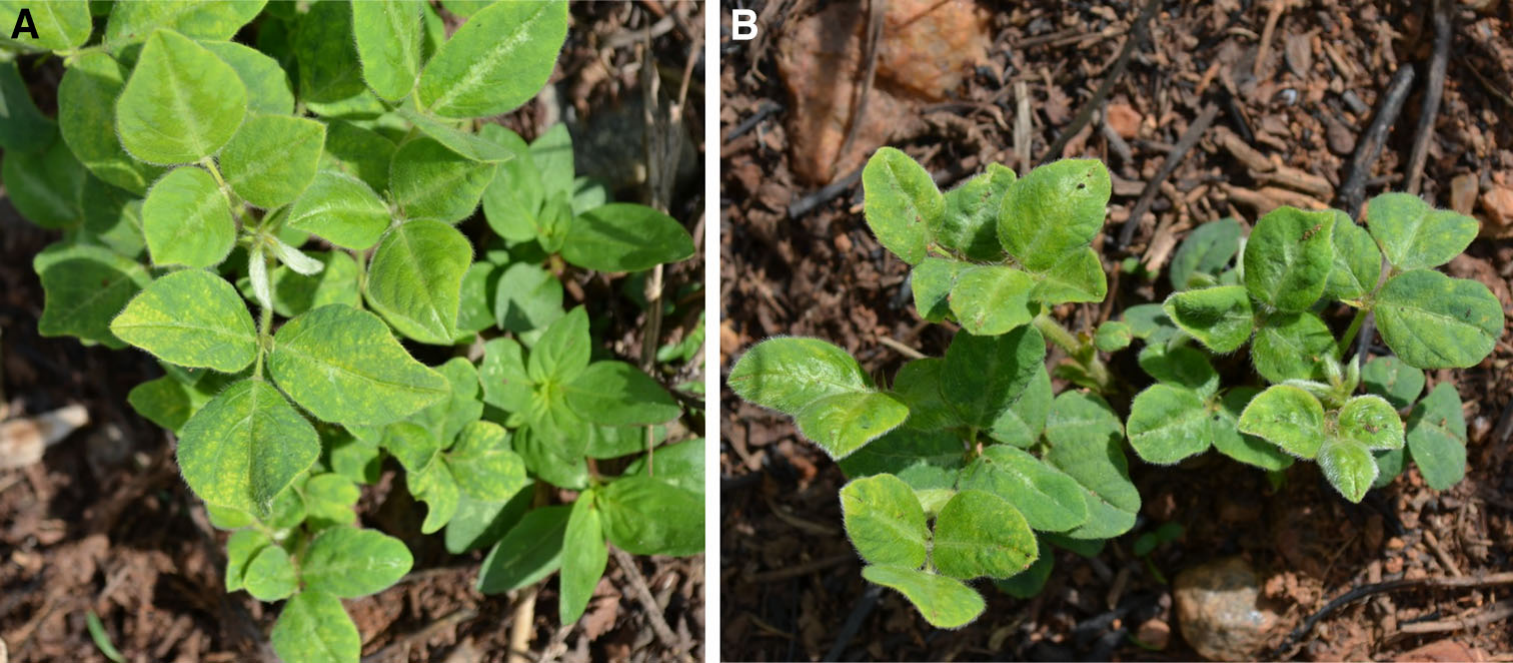
*Desmodium* sp. plants analyzed in this work showing mottle symptoms on leaves. (A) sample UG4, (B) sample UG5

Total nucleic acids were extracted from leaf samples using a modified CTAB method [22]. To test for the presence of begomoviruses in the samples, putatively causing the observed symptoms, nucleic acids were used as a template for rolling-circle amplification (RCA) using φ29 DNA polymerase (TempliPhi kit, GE Healthcare**)** and amplified RCA products were digested with a set of restriction enzymes (ClaI, BamHI, EcoRI, HindIII, NcoI, NheI and SalI) [17]. Putative full length begomoviral genomic components (~2.8 kbp) were cloned from each sample (EcoRI and HindIII for UG4 and ClaI and EcoRI for UG5) into pBlueScript II SK (+) (Stratagene) and selected clones (one per sample and restriction enzyme) were sequenced at Macrogen Inc. (Seoul, South Korea). Sequences were assembled with SeqMan software included in the DNASTAR package (DNASTAR Inc.). Open reading frames (ORFs) were identified using Open Reading Frame Finder (NCBI) and confirmed using the BLAST program (https://blast.ncbi.nlm.nih.gov/Blast.cgi) on their deduced amino acid sequences. Initial sequence identity comparison was performed using the BLAST program, sequences were aligned with MUSCLE [9] and pairwise identity scores were calculated using SDT (Sequence demarcation tool) [20]. A phylogenetic analysis using maximum likelihood (ML) was used after selecting the best-fit model of nucleotide substitution based on corrected Akaike Information Criterion (AICc) and Bayesian Information Criterion (BIC) as implemented in MEGA 6 [25]. Recombination analysis was performed using RDP4 [18] after alignment, with MUSCLE, of the sequences selected with SWeBLAST (with a window size of 200 and a step size of 200) [12]. SWeBLAST avoids the significant problem of deciding which sequences to compare, thus allowing identification of putative parents of recombinant sequences (four sequences for DNA-A and nine sequences for DNA-B). Only recombination events detected with at least five methods with p-values lower than 10^−2^ were considered.

The cloned genome components from each sample were shown to correspond to begomoviral DNA-A and DNA-B components. DNA-A component from sample UG4 (2767 nt, EcoRI fragment, KY294724) and UG5 (2767 nt, ClaI fragment, KY294725) showed the highest nucleotide sequence identity (79.9% and 80%, respectively) to SbMMoV (GQ472984), a legumovirus found in soybean (*Glycine max*) in Nigeria [1]. The DNA-B component from sample UG4 (2715 nt, HindIII fragment, KY294726) exhibited the highest nucleotide sequence identity (65.4%) with an isolate of African cassava mosaic virus (ACMV) (KJ887741) from Madagascar [8], while UG5 DNA-B (2713 nt, EcoRI fragment, KY294727) exhibited the highest nucleotide sequence identity (66.4%) with another isolate of ACMV (HE616778) from Burkina Faso [26]. Pairwise nucleotide identities between DNA-A and DNA-B from samples UG4 and UG5 were 100% and 99.7%, respectively, confirming that the virus identified from both samples belonged to the same begomovirus species. Also, the virus showed a typical genome organization of Old World bipartite begomoviruses. In accordance, therefore, with current taxonomic guidelines for the genus *Begomovirus* (a new DNA-A sequence with less than 91% pairwise identity to any other published begomovirus DNA-A sequence will belong to a new begomovirus species) [5], the isolates described here ([Uganda-Kikonge UG4-2015] and [Uganda-Kikonge UG5-2015]) represent a novel species for which we propose the name Desmodium mottle virus (DesMoV).

DNA-A and DNA-B from both samples showed a CR of 179 nt (DNA-A) and 156 nt (DNA-B) with sequence identities of 90.2% (sample UG4) and 90.8% (sample UG5). The difference in length of the CR is due to a deletion in DNA-B. Both components from each sample showed three copies of iterons (AATCGGGGGT) (one is inverted and the most proximal to TATA box is imperfect), indicating that DNA-A and DNA-B isolated from each sample constitute a cognate pair.

A phylogenetic tree based on alignment of the DNA-A sequences obtained here with those of selected begomoviruses (including one sequence from each legumovirus species) showed that they grouped in a cluster with the legumoviruses (Fig. 2A). However, DNA-Bs grouped with ACMV, a typical OW begomovirus (Fig. 2B). Similar phylogenetic relationships have been described previously for SbCBV, the only bipartite legumovirus identified in Africa until now [1]. This is an example of the distinct evolutionary history undergone by the DNA-A and DNA-B genome components, as shown previously for other begomoviruses [3, 6, 11, 23, 24]. No recombination event was detected in any of the genome components of DesMoV.

**Fig. 2:**
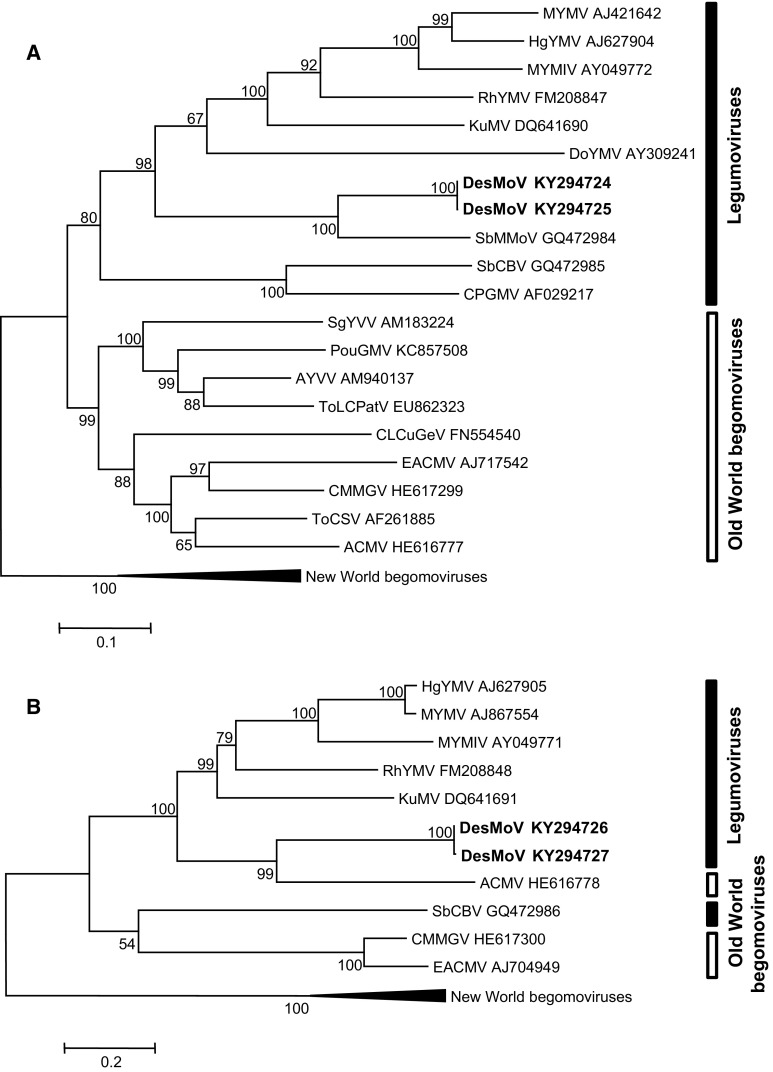
Phylogenetic trees illustrating the relationship of isolates of Desmodium mottle virus (DesMoV) DNA-A (A) and DNA-B (B) to other begomoviruses. The trees were constructed by the maximum-likelihood method (1000 replicates) with the MEGA 6 program using the best fit model, TN93+G+I for DNA-A and HKY+G+I for DNA-B. ACMV, African cassava mosaic virus; AYVV, Ageratum yellow vein virus; CLCuGeV, cotton leaf curl Gezira virus; CMMGV, cassava mosaic Madagascar virus; CPGMV, cowpea golden mosaic virus; DoYMV, Dolichos yellow mosaic virus; EACMV, East African cassava mosaic virus; HgYMV, horsegram yellow mosaic virus; KuMV, kudzu mosaic virus; MYMIV, mungbean yellow mosaic India virus; MYMV, mungbean yellow mosaic virus; PouGMV, Pouzolzia golden mosaic virus; RhYMV, Rhynchosia yellow mosaic virus; SbCBV, soybean chlorotic blotch virus; SbMMoV, soybean mild mottle virus; SgYVV, Siegesbeckia yellow vein virus; ToCSV, tomato curly stunt virus; ToLCPatV, tomato leaf curl Patna virus. A set of New World begomoviruses was used as the outgroup. The bar below each tree indicates nucleotide substitutions per site

DesMoV is the first legumovirus to be described from East Africa to date and because it is phylogenetically closely related to begomoviruses that infect soybean or cowpea in West Africa, it may represent a potential threat to these crops. Additional research work to investigate its host-plant range and whitefly transmission characteristics, therefore, should be initiated to assess the threat to crops posed by this newly discovered begomovirus.
